# Prediction ability of genome-wide markers in *Pinus taeda* L. within and between population is affected by relatedness to the training population and trait genetic architecture

**DOI:** 10.1093/g3journal/jkab405

**Published:** 2021-11-25

**Authors:** Edwin Lauer, James Holland, Fikret Isik

**Affiliations:** 1 Department of Forestry and Environmental Resources, North Carolina State University, Raleigh, NC 27695, USA; 2 USDA-ARS Plant Science Research Unit, North Carolina State University, Raleigh, NC 27695, USA

**Keywords:** QTL, loblolly pine, linkage disequilibrium, genomic relationships, cloned progeny tests, cross-validation, GBLUP, GWAS, Genomic Prediction, GenPred, Shared Data Resource

## Abstract

Genomic prediction has the potential to significantly increase the rate of genetic gain in tree breeding programs. In this study, a clonally replicated population (*n* = 2063) was used to train a genomic prediction model. The model was validated both within the training population and in a separate population (*n* = 451). The prediction abilities from random (20% *vs* 80%) cross validation within the training population were 0.56 and 0.78 for height and stem form, respectively. Removal of all full-sib relatives within the training population resulted in ∼50% reduction in their genomic prediction ability for both traits. The average prediction ability for all 451 individual trees was 0.29 for height and 0.57 for stem form. The degree of genetic linkage (full-sib family, half sib family, unrelated) between the training and validation sets had a strong impact on prediction ability for stem form but not for height. A dominant dwarfing allele, the first to be reported in a conifer species, was discovered via genome-wide association studies on linkage Group 5 that conferred a 0.33-m mean height reduction. However, the QTL was family specific. The rapid decay of linkage disequilibrium, large genome size, and inconsistencies in marker-QTL linkage phase suggest that large, diverse training populations are needed for genomic selection in *Pinus taeda* L.

## Introduction

Forest trees are slow growing, long-lived species that require multiple years to reach reproductive maturity (Namkoong *et al.* 2012). Traits of economic interest in a breeding program require many years of growth prior to measurement to yield reliable phenotypic information (Isik and McKeand 2019). These attributes lengthen the generation time and reduce the rate of genetic gain from tree breeding programs (Grattapaglia and Resende 2011). As in cattle breeding, forward selection of young untested individual progeny is an area of intense research interest in tree breeding since this method reduces the time of a breeding cycle and has a direct impact on the rate of genetic gain (Falconer and Mackay 1996; Lillehammer *et al.* 2011).

In the conifer *Pinus taeda* L., the breeding cycle begins with a controlled cross between two individuals in a breeding orchard. After 18 months of cone maturation, seeds are extracted and used for progeny test establishment. These progeny tests are measured 4–5 years after planting. Traditionally, individual tree breeding values are predicted with pedigree-based best linear unbiased prediction (ABLUP) (Mrode 2014) and selection decisions are made using a multi-trait index incorporating growth, stem form, and disease resistance traits. Selection candidates are then top grafted into mature breeding orchard trees and are available for breeding in 3 years (Isik and McKeand 2019). If genomic selection (GS) could be used to select individual trees at the seedling stage, the field-testing phase could be eliminated or reduced. This would reduce the cycle time by 4–5 years and double of the rate of genetic gain (Isik 2014).

Many proof-of-concept studies using real datasets have been conducted to examine factors related to the success of GS in forest trees (Resende *et al.* 2012b; Beaulieu *et al.* 2014; Isik *et al.* 2016; Ukrainetz and Mansfield 2020). In the first report of GS in a tree species (Resende *et al.* 2012a), two unrelated clonal populations of Eucalyptus were genotyped with a set of 3000 DArT markers. Accuracy of GS, assessed through “leave-one-out” cross-validation was found to closely match that of phenotypic selection but the prediction ability dropped significantly when one population was used to predict another (Resende *et al.* 2012a). Similar results were observed for a clonal population of loblolly pine (Resende *et al.* 2012b); in that study, GS accuracies from random cross-validation ranged between 0.63 and 0.75 for growth. In white spruce, a maternal half-sib population of 1694 trees from 214 parents was genotyped with a set of 6385 SNP markers (Beaulieu *et al.* 2014). Accuracy of GS for multiple traits varied between 0.33 and 0.44 when half-sib relatives of the prediction set were present in the training set. When half-sib relatives were removed from the training population, GS accuracies were reduced by more than 50% for most traits (Beaulieu *et al.* 2014). In another study in maritime pine, a two-generation dataset composed of 661 trees genotyped with 2500 markers provided predictive abilities between 0.4 and 0.5 for several traits in two random cross-validation scenarios (Isik *et al.* 2016). Both cross-validation scenarios used in that study were random cross-validation, differing only in training set population size.

Most of the proof-of-concept studies of GS in tree species utilized random cross-validation schemes that included familial relatives of the prediction set in each training set. Since the loblolly pine breeding program managed by the Cooperative Tree Improvement Program at North Carolina State University utilizes a full-sib mating design with many crosses produced each year (Isik and McKeand 2019), a realistic application for GS would involve prediction of breeding values within new full-sib families not represented in the training population. Within-family selection requires accurate estimation of Mendelian sampling effects to rank individuals (Werner *et al.* 2020). Because the earlier literature on GS in tree species utilized random cross-validation, they were not explicitly testing the ability of markers to predict Mendelian sampling effects. Random cross-validation tests the ability of markers to predict total breeding value, which may provide higher accuracies than stratified sampling schemes that withhold close relatives of the test set from the training set (Werner *et al.* 2020). Studies in other species including maize and triticale suggest that family structure between training and prediction sets is a key determinant of GS prediction ability (Massman *et al.* 2013; Lehermeier *et al.* 2014; Wurschum *et al.* 2017). Since most tree GS studies utilized random cross-validation, there is a knowledge gap around the best way to implement GS in conifer breeding. In this study, we compare the relative ability of genome-wide markers to predict total breeding value and Mendelian sampling effects through two cross-validation scenarios designed to address among-family and within-family selection separately.

### Objectives

The first objective of this study was to measure the ability of a clonal training population to predict genomic estimated breeding values (GEBV) of individual trees in a separate population. The second objective was to study the effect of genetic similarity between the training and validation population on prediction accuracy. We genotyped a number of trees from wide-ranging progeny tests with varying degrees of relationship with the clonal training population. Genetic architectures for stem height and straightness (a categorical trait) were studied through individual trait genome-wide association studies (GWAS), and significant markers were tested as fixed-effect covariates in GBLUP models. Genome-wide analysis of linkage disequilibrium (LD) was conducted to contextualize the modeling results and develop recommendations for the practical implementation of GS in conifer breeding programs.

## Materials and methods

### Clonal training population

The population used for model training, Atlantic Coastal Elite (ACE), is a clonally replicated collection of full-sib families established across eight environments in the early 2000s (Shalizi and Isik 2019). A total of 24 parents was intermated in three disconnected eight-parent diallels, producing 76 full-sib families. Seedlings from these families were screened for fusiform rust disease, caused by *Cronartium quercuum* (Berk.) Miyabe ex Shirai f.sp. *fusiforme* at the USDA Forest Service Resistance Screening Center in Asheville, NC using an artificial inoculation. From the original set of 76 full-sib families, 25 were removed due to high susceptibility to disease. This resulted in the removal of three parents from the original disconnected diallel mating design.

An average of 46 full-sib progeny from each of the remaining 51 families was vegetatively propagated. From each progeny, eight rooted cuttings (ramets) were produced. These ramets were established in eight environments across the southeast United States (Shalizi and Isik 2019). Each environment featured an alpha-cyclic row–column incomplete block design with a single replication. During test establishment, dead seedlings were replaced with seedlings from an open-pollinated half-sib family from a single parent. Additionally, seven cloned open-pollinated families from parents not used in the disconnected diallels were established in the eight tests.

Tests were measured 6 years after establishment. Height was measured in units of feet and was converted to meters. Stem form was recorded as an ordinal variable with values from 1 to 6, with 1 being the straightest. At the time of measurement, a total of 2499 clonal genotypes were present across the eight tests. These genotypes were members of 51 full-sib families and 7 open-pollinated families.

### Fourth cycle progeny tests for model validation

In order to test the ability of GS models trained within a clonal population to predict GEBVs of individual trees in a separate population, a group of trees with both genotypic and phenotypic records were required. These trees were sampled from a set of 18 progeny tests from the fourth Cycle Coastal breeding population of The North Carolina State University Cooperative Tree Improvement Program (Isik and McKeand 2019). Each location was established using an alpha-cyclic incomplete row–column experimental design with between five and 10 replications. Each location was established with a collection of full-sib, open-pollinated, and checklot trees from bulked seedlots. The full-sib progenies were derived from crosses prescribed by the mating design algorithm implemented in MateSelect software (Kinghorn 2011).

The average number of shared parents between pairs of fourth cycle progeny tests was 100.5, representing 72% of all parents tested within any given site. The connectivity at the level of full-sib families was 59.2 for pairs of fourth cycle tests, representing 51% of all full-sib families at any given site. The fourth cycle progeny tests were connected to the ACE clonal tests through an average of 18.6 shared parents (Supplementary Figure S1). Connectivity between the ACE clonal tests (training population) and the fourth cycle tests (validation population) at the level of full-sib families was lower, with only two full-sib families appearing in both sets.

All fourth cycle tests were measured 4 or 5 years after establishment for the same traits measured in the ACE clonal tests. A set of 451 individual trees was sampled from the fourth cycle tests for testing the ability of GS models to predict individual tree GEBV. In the set of individual samples, 52 were members of a full-sib family that was also represented in the ACE training population, 213 trees were members of seven half-sib families that shared one parent in common with the ACE training population, and 186 had no direct parental relationships with the training population. Each half-sib family contained two or more nested full-sib families.

### Genotyping and SNP filtering

A total of 2063 clones within the ACE training population were genotyped with the Pita50K Affymetrix microarray (Caballero *et al.* 2021). From the fourth-Cycle progeny tests, 451 individual trees were genotyped. Genotypes were called from raw microarray data using Life Science’s proprietary software Axiom Analysis Suite (*Axiom Analysis Suite v5.1* 2020). All manipulation of SNP genotype data was performed using customized scripts in the R programming environment (R Core Team 2021). Various functions within the *dplyr* and *ggplot2* packages were used extensively throughout this work (Wickham 2014). Before combining the genotyping data from the two populations, any SNP that were monomorphic in either population were removed. SNP filtering was then applied to the combined genotype dataset. Markers were removed for two reasons: (1) having a minor allele frequency < 0.01 or (2) having an observed heterozygote frequency deviating more than 15% from the Hardy–Weinberg expectation (Wiggans *et al.* 2009). This resulted in the retention of 29,135 markers. Samples with more than 10% missing data were removed. The final proportion of missing genotype data was 2.5%.

### Genotype imputation and calculation of G

The genotype matrix (2514, 29135) was used for two purposes: GWAS and GBLUP. For GBLUP, all 2063 ACE clonal genotypes plus 451 individual trees sampled from fourth-Cycle progeny tests were used. The marker matrix used in GBLUP will be referred to as **M1**. For GWAS, only the markers mapped in the Lauer and Isik (2021) consensus map were retained, resulting in a (2514, 8523) matrix. The marker matrix used in GWAS will be referred to as **M2.**

The genomic relationship matrix **G** was calculated from **M1** using the R package AGHMatrix (Amadeu *et al.* 2016), using Method 1 described by VanRaden (2008). Missing data were imputed using the mean genotype for each marker. The matrix **G** is shown as a heatmap in Supplementary Figure S3. For **M2**, the genetic map was used for phasing and imputation with Beagle v5.1 (Browning *et al.* 2018). After phasing and imputation, all samples that were not part of the ACE clonal population and all SNP with a minor allele frequency <0.01 were removed from **M2**, resulting in a (2063, 8437) matrix for use in GWAS.

### Statistical analyses

All linear mixed models used for the purpose of BLUE and BLUP estimation were implemented in ASReml 4.1 (Gilmour *et al.* 2015). The linear mixed model used for GWAS was implemented in the R package rrBLUP (Endelman 2011). Genomic relationship matrix for genotyped individuals from ACE1 and fourth Cycle progeny tests is presented as a heatmap using the R package *popkin* (Ochoa and Storey 2021).

A linear mixed model was used to obtain spatially adjusted pseudo-phenotypes (the best linear unbiased estimates, BLUE) for the ACE clonal population for GWAS. The model was:

(Eq. 1) y=Xβ+Zu+ ε,

where **y** was a response vector for 14,857 vegetative ramets of 2,494 clones. X is a (14857, 2503) design matrix mapping the records in **y** to the population mean, eight test sites, and 2494 clones. The vector of fixed effects β contains the population mean, the effects for the eight ACE clonal sites, and fixed effects for the 2494 clones. The matrix **Z** is a (14857,878) design matrix for random effects, mapping the records in **y** to 360 column-within-test effects, and 518 row-within-test effects. The random effects vector u contains 360 column-within-test effects and 518 row-within-test effects. The random error vector ε contains 14,857 residuals.

The assumptions of the model were that y ∼ MVN(Xβ, V) where $V=\;\left[\begin{array}{c} u\\ e \end{array}\right]=\;\left[\begin{array}{cc} G & 0\\ 0 & R \end{array}\right]$. The variance-covariance for the random effects u were block-diagonal with a separate variance for each test. The variance-covariance matrix for the residuals ε at each location was a separable first-order autocorrelation matrix. These submatrices were combined as a direct sum to form the residual matrix **R**.

(Eq. 2) $$R=\;{⊕}_{t=1}^{8}[\sigma_{e\left(t\right)}^{2}\Sigma_{r\left(t\right)}\left(\rho_{r\left(t\right)}\right)⊗\Sigma_{c(t)}\left(\rho_{c(t)}\right)],$$

where $\sigma_{e\left(t\right)}^{2}$ is the residual variance for test *t*, $\Sigma_{r\left(t\right)}\left(\rho_{r\left(t\right)}\right)$ is the [r, r] correlation matrix for the rows of test *t*, and $\Sigma_{c(t)}\left(\rho_{c(t)}\right)$ is the [c, c] correlation matrix for the columns of test *t*.

### Genome-wide association analysis (GWAS)

The *GWAS* function of the R package rrBLUP was used as a mixed-model platform for association analysis. The following linear mixed model was fit for each marker:

(Eq. 3) $$y=X\beta +Z_{g}+\;S_{\tau }+\;\varepsilon ,$$

where **y** represents a vector (2063,1) of pseudo-phenotypes [the BLUE of genotyped ACE clones estimated in (Eq. 1)]. X is a (2063,3) design matrix relating the BLUE in **y** to the loadings for each clone on the first three principal components of the kinship matrix, represented in this model by matrix β. The design matrix for the random polygenic effects, Z, related the BLUE in **y** to the polygenic effects in **g**. Finally, the fixed effect of each marker was obtained using a design matrix S taking on values of –1, 0, and 1 for the major-allele homozygote, heterozygote, and minor-allele homozygote, respectively. The residual errors for 2063 clones were contained in ε. The random polygenic effect had the expectations $g\;∼\;N(0,K\sigma^{2})$ with K representing the realized genomic relationship matrix (VanRaden 2008). The residual errors were assumed to have a normal distribution with the expectations $\varepsilon \;∼\;N(0,\;I\sigma_{e}^{2})$. The loadings in β were determined from spectral decomposition of K matrix using the base R function *eigen* (Endelman 2011), and were incorporated into the model to account for large-scale population structure.

To estimate family-specific effects for significant GWAS associations, simple marker regression was performed within families displaying genotypic variation at the significant marker. The model applied to each family was:

(Eq. 4) y=Xb+ε,

with **y** representing the height BLUE estimated from the model in Equation (1) for a single full-sib family. The design matrix **X** is a vector of 0’s and 1’s, representing homozygous and heterozygous genotypes at the significant marker, while **b** represents the regression coefficient (the average effect). Here, ε represents the random residual error, which in this case was assumed ∼iid(0, $I\sigma_{\varepsilon }^{2}$).

### Animal model for prediction of estimated breeding values

Univariate animal models were used to predict breeding values for all genotypes in the ACE and fourth-Cycle populations. The animal model took the form:

(Eq. 5) $$y=X\beta +Z_{1}r+Z_{2}u+\varepsilon ,$$

with **y** being a vector (27883,1) of phenotypic records (height or straightness) for 14,857 trees (vegetative ramets) in ACE clonal tests and 13,026 individual trees in fourth-Cycle. Matrix **X** is a (27883,19) design matrix mapping the fixed effects in β vector to the response vector **y**. The fixed effects vector β included the population mean and the test effects for the eight ACE clonal tests and 18 tests of fourth-Cycle population. The design matrix for the random experimental design effects **Z_1_** had dimensions (27883, 3303). The random experimental design effects vector, r, included 100 replication-within-test effects for the fourth-Cycle tests, 1103 column-within-replication effects for fourth-Cycle tests, 1222 row-within-replication effects for fourth-Cycle tests, 360 column-within-test effects for the ACE clonal tests, and 518 row-within-test effects for the ACE clonal tests. The design matrix for the genetic effects, **Z_2_**, had dimensions (27883, 403575). The vector of random genetic effects, u, included 315 specific combining ability effects and 403,260 additive genetic effects for the 15,510 entries in the pedigree across the 26 test sites. The random residual vector was ε. The variance–covariance matrix for the residuals, **R**, was block-diagonal with a separate residual variance for each location.

The assumptions of the model were that y ∼ MVN(Xβ, V) where $=\;\left[\begin{array}{c} r\\ u\\ e \end{array}\right]=\;\left[\begin{array}{ccc} G_{r} & 0 & 0\\ 0 & G_{u} & 0\\ 0 & 0 & R \end{array}\right]$. The variance–covariance matrix for the random effects, **G**, had two sub-matrices $G_{r}$ and $G_{u}$. The variance–covariance matrix for the random experimental design effects, $G_{r}$, was formed as a direct sum: $G_{r}=I_{100}\sigma_{\mathrm{rep}}^{2}⊕I_{1103}\sigma_{cr}^{2}⊕I_{1222}\sigma_{rr}^{2}⊕I_{360}\sigma_{ct}^{2}⊕I_{518}\sigma_{rt}^{2}$, where the variance parameters for reps within fourth-Cycle tests, columns within reps for fourth-Cycle tests, rows within reps for fourth-Cycle tests, columns within tests for ACE clonal tests, and rows within tests for ACE clonal tests are indexed by the subscripts “*rep*,” “*cr*,” “*rr*,” “*ct*,” and “*rt*,” respectively. The variance–covariance matrix for the random genetic effects, $G_{u}$, was a direct sum: $G_{u}=I_{315}\sigma_{\mathrm{SCA}}^{2}⊕\left[(11_{26}^{`}\sigma_{u}^{2}+\;I_{26}\sigma_{u.e}^{2})⊗A\right]$ where the variance parameters for the SCA variance, genetic variance, and genotype-by-environment variance are indexed by “*SCA*,” “*u*,” and “*u.e*,” respectively. In this formulation (CORUV structure in ASReml software syntax), a single variance parameter is estimated for genotype-within-environment effects, and a single covariance is estimated for among-environment genetic covariance. These parameters are interpreted as $\left(\sigma_{u}^{2}+\sigma_{u.e}^{2}\right)$ and $\sigma_{u}^{2}$, respectively. The numerator relationship matrix **A** had dimensions of (15510, 15510).

Marginal estimated breeding values (EBV) were obtained by averaging the genotype-within-environment effects for each genotype over 26 tests: ${\hat{u}}_{i}=\frac{1}{26}\sum_{t=1}^{26}{\hat{u}}_{i(t)}$. The term ${\hat{u}}_{i(t)}$ represents the additive genotype-within-environment effect for the i^th^ individual within the t^th^ environment. Reliabilities were computed using the prediction error variance (PEV) of each BLUP: $r^{2}=1-\;\frac{\mathrm{PEV}}{\sigma_{u}^{2}(1+F)}$. The F term represents the inbreeding coefficient, estimated as $F_{i}=A_{\mathrm{ii}}-1$ where $A_{\mathrm{ii}}$ is the diagonal value of the additive relationship matrix for the i^th^ individual.

### Genomic BLUP

To test the ability of genome-wide markers to predict additive genetic merit of trees, a GBLUP model was applied to the same dataset that was used for the estimation of BLUE of the ACE clonal population, excluding non-genotyped clones. In addition, 451 rows were appended to the bottom of the dataset representing the genotyped fourth-Cycle trees, but their phenotypic data were not included in the model. The mixed linear model for GBLUP can be written as before:

(Eq. 6) y=Xβ+Zu+ ε,

here, **y** represents the response vector for 12223 ramets (genetically identical copies of 2063 clones) and the fourth cycle population 451 individual trees. No phenotypic data were included for the individual trees. The design matrix for the fixed effects X had dimensions (12664, 9). The vector of fixed effects in β included the population mean and eight test effects. The design matrix for the random effects Z had dimensions (12664, 5906). The random effects vector u contained 360 row-within-test effects, 518 column-within-test effects, 2514 additive genetic effects, 2514 non-additive genetic effects, and 20112 genotype-by-environment effects. The vector of random residuals was **ε**.

The variance–covariance matrix for the additive genetic effects was $\sigma_{u}^{2}G$, with **G** representing the realized relationship matrix calculated from SNP markers using Method 1 from VanRaden (2008). The variance–covariance for the non-additive genetic effects was identity, $\sigma_{\mathrm{na}}^{2}I_{2514}$. In this context, the non-additive component contains dominance, higher-order epistatic terms, effects of major genes, and any other genetic effects that do not fit the additive genomic relationships. The variance–covariance for the genotype-by-environment term was $I_{20112}\sigma_{\mathrm{ue}}^{2}$. The variance–covariance matrix for the residuals was block-diagonal with a unique residual variance for each location as explained for Equation (3). Reliabilities for each GEBV were computed using the same method as the animal model, with inbreeding coefficients estimated from **G** instead of **A**.

A significant GWAS association on linkage Group 5 for tree height was used as a fixed-effect covariate in GBLUP. The average effect of the allele substitution (α) for the QTL on linkage Group 5 was estimated using the same GBLUP model described above, with an additional covariate in X taking values of 0 and 1 for the major-allele homozygote and heterozygote, respectively. The average effect was estimated as the regression coefficient for the covariate in β.

### Heritability

To assess the ability of genome-wide markers to capture additive genetic variation relative to standard ABLUP, narrow-sense heritability was estimated from model Equation (4):

(Eq. 7) $$h^{2}=\;\frac{{\hat{r}}_{a}\sigma_{u}^{2}}{\sigma_{u}^{2}+\sigma_{\mathrm{SCA}}^{2}+\frac{1}{t}\left(\sum_{t=1}^{t}\sigma_{e\left(p\right)}^{2}\right)},$$

where ${\hat{r}}_{a}$ is the common genetic correlation between sites, $\sigma_{u}^{2}$ is the genotype-within-environment variance, and *t* is the number of fourth-Cycle progeny tests. Since the objective was prediction of individual tree breeding values, the residual term $\frac{1}{t}\left(\sum_{t=1}^{t}\sigma_{e\left(p\right)}^{2}\right)$ is the average residual variance of the fourth-Cycle progeny tests.

Heritability of clone means was estimated in the same manner, except that the residual term was weighted by the harmonic mean number of vegetative ramets per clone (Holland *et al.* 2003). The heritability of clone means could be written as:

(Eq. 8) $$h_{c}^{2}=\frac{{\hat{r}}_{a}\sigma_{u}^{2}}{\sigma_{u}^{2}+\sigma_{\mathrm{SCA}}^{2}+\frac{\sum_{c=1}^{c}\sigma_{e(c)}^{2}}{\mathrm{nc}}},$$

where *n* is the harmonic mean number of ramets per clone, and *c* is the number of clonal tests. The mean reliability of genotyped trees was compared between the animal model and GBLUP to assess the impact of information loss caused by removal of the pedigree and phenotypic data. Standard errors of heritabilities were estimated using the Delta method in ASReml 4.1 (Gilmour *et al.* 2015).

Heritability of family means for fourth-Cycle tests was estimated using the following formula (Isik *et al.* 2017):

$$h_{f}^{2}=\frac{{\hat{r}}_{a}\sigma_{u}^{2}}{\frac{\sigma_{u}^{2}}{s}+\frac{(1-s){\hat{r}}_{a}\sigma_{u}^{2}}{s}+\frac{\sum_{t=1}^{t}\sigma_{e\left(p\right)}^{2}}{rs}},$$

where *s* is the total number of fourth-Cycle progeny test sites and *r* is the harmonic mean number of progeny per parent.

To estimate missing heritability (de los Campos *et al.* 2015), average reliabilities ($r_{n}^{2}$) for clonal genotypes with phenotypic data were compared between ABLUP and GBLUP. In the case of unbalanced data, the response to selection may vary depending on the selection unit since the amount of information in the model is not the same for each genotype (Piepho and Möhring 2007). A generalized heritability taking into account the heterogeneous information content for Q genetic effects in the model is the mean reliability:

(Eq. 9) $${\bar{r}}^{2}=\;Q^{-1}\sum_{n=1}^{N}r_{n}^{2}.$$

Conifer genetic trials typically constitute sets of full-sib families derived from crosses between unrelated individuals, so F is usually 0. The average reliability $E(r^{2})$ of individual BLUPs is analogous to the classical heritability from balanced completely randomized designs with independent genetic effects (Piepho and Möhring 2007):

(Eq. 10) $$E\left(r^{2}\right)=\;\frac{\mathrm{cov}(u_{i},{\hat{u}}_{i})}{\sqrt{\sigma_{u}^{2}\sigma_{\hat{u}}^{2}}}=\frac{\sigma_{u}^{2}}{\sigma_{P}^{2}}=h^{2}.$$

To estimate missing heritability for each trait ($h_{m}^{2})$, average reliabilities from ABLUP and GBLUP for clonal genotypes were substituted for the $h^{2}$ and $h_{g}^{2}$ terms, respectively (de los Campos *et al.* 2015):

(Eq. 11) $$h_{m}^{2}=\frac{h^{2}-h_{g}^{2}}{h^{2}}.$$

### Cross-validation

Two cross-validation scenarios were used to test the ability of genome-wide markers to predict breeding values. The first was random fivefold cross validation (Random-CV) within the ACE clonal population. In each of 10 replications of Random-CV, the phenotypic data for a random set of 20% of the ACE clonal genotypes was held out of the model, and GEBV for these clones were predicted using the other 80% of the population. Since the sampling was random, the training population for each clone was a mixture of full-sib, half-sib, and distant relatives.

The second cross-validation scenario was full-sib cross validation (Fullsib-CV). In this scenario, each of 51 full-sib families in the ACE population was held out of the model in turn, and predicted using the other 50 families. This scenario represents a common breeding situation in which a new full-sib family is produced that is not represented in the training population. In this scenario, the training population for each clone lacked full-sib relatives, but contained a mixture of half-sib and more distantly related relatives.

In each replication of cross-validation, variance parameters were fixed using estimates from the full model (with all ACE clonal data). Since the breeding program conducts forward selection using individual tree BLUP (EBV), prediction ability in each fold was measured as $\hat{r}\left(\mathrm{EBV},\;\mathrm{GEBV}\right)$, the Pearson correlation coefficient between EBVs predicted from the ABLUP animal model, and GEBVs predicted from GBLUP.

Since the fourth-Cycle progeny tests were not included in the training model for GBLUP, cross-validation could not be replicated for the 451 genotyped trees. The impact of relatedness on prediction ability was assessed using the full GBLUP model trained with all ACE clones, and prediction ability was measured as the correlation between their GEBV from GBLUP and their EBV from ABLUP.

### Linkage disequilibrium

Genome-wide analysis of LD was conducted for genotype matrix **M2** using the R package *pegas* (Paradis 2010). The function “*LDScan*” was used to estimate interallelic R^2^ values for all pairs of SNPs on each linkage group using the phased genotypes in **M2**. The rate of LD decay over genetic distance was estimated by averaging the R^2^ values for all pairs of SNP within 0.5 cM bins of map distance from 0 to 200 cM. The rate of LD decay over physical distance was estimated in the same manner, except that only pairs of markers occurring on the same contig of the Pitav2.01 reference genome were utilized in the analysis.

## Results

### Marker-trait associations

**Figure 1:**
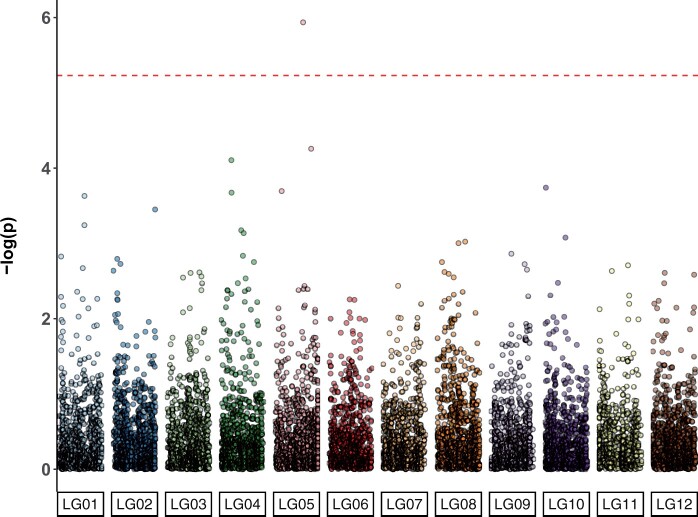
Significance levels (log scale) of 8437 SNPs distributed across 12 linkage groups for association with height in *Pinus taeda*. The Bonferroni-adjusted-log_10_ (*P*-value) for an experiment-wise Type I error rate of 0.05 is shown as a horizontal red dashed line. The significant marker, PitaSNP287174, was located at 166.9 cM on linkage Group 5. The average effect of the marker was –0.34 m, and its minor allele frequency was 0.02. The marker segregated in four full-sib families within ACE1 population.

**Figure 2:**
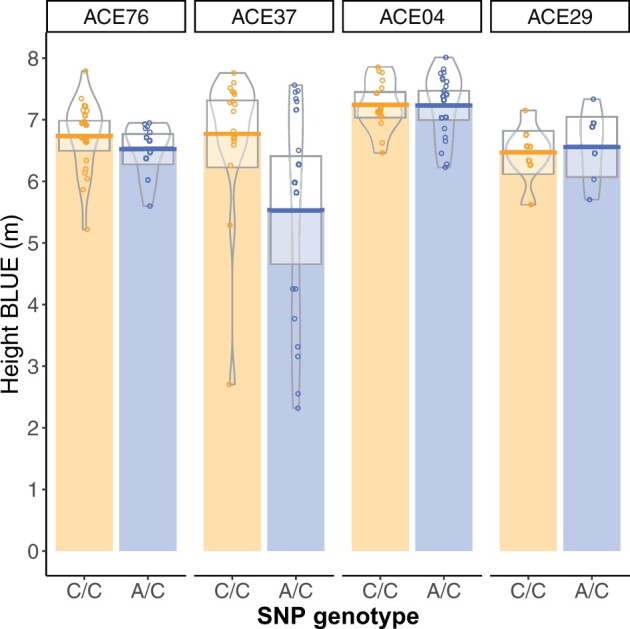
The effect of significant SNP PitaSNP287174 on linkage Group 5 on height varied across four families, suggesting variation in linkage phase between this marker and a linked QTL. Phenotype distributions for height (m) for the two genotypic classes within the four families segregating at the marker are shown here. Full-sib families ACE76 and ACE37 share one common parent. The effect of the marker was statistically significant only in family ACE37 (Supplementary Table S1).

A significant association was observed for tree height on linkage Group 5 at 166.9 cM at marker PitaSNP287174 (Figure 1), and no significant associations were observed for stem form (data not shown). The QQ-plot suggested a close agreement to the null hypothesis for the majority of SNP (Supplementary Figure S2). The minor allele at PitaSNP287174 was present at a frequency of 0.02 and had an average effect of –0.34 m, which was the average difference between trees with 0 copies of the minor allele and 1 copy. No minor-allele homozygotes were observed in the population. Genotypic values were inspected in the four full-sib families segregating at the marker (Figure 2). Marker PitaSNP287174 showed evidence for the segregation of a large-effect dwarfing gene in two full-sib families (ACE76, ACE37), which shared one parent. The SNP was not associated with any effect on height in two other families in which it was segregating, and which did not derive from the parent shared by families ACE76 and ACE37. This suggests that the marker-QTL linkage phase varied across families. The height reducing effect was observed in two of the four families, but was statistically significant only in family ACE37 (Supplementary Table S1). Since the minor allele was associated with a height decrease in the heterozygous condition, this represents the first dominant dwarfing allele reported in a conifer species.

### Genetic parameters and reliabilities of predictions

Type-B genetic correlations of genotypic values across environments were 0.79 and 0.87 for height and stem form, respectively (Table 1), suggesting limited genotype-by-environment interaction. The traits had similar clone-mean heritability estimates (0.49 and 0.51), but the narrow-sense heritability for stem form (0.19) was far lower than for height (0.31). Heritability of family means was > 0.9 for both traits (Table 1). Reliabilities of EBV for clones were ∼ 0.68 for both height and stem form. For individual trees, reliabilities were close to 0.55 for both traits, with stem form showing slightly lower estimates than height (not shown).

**Table 1:** Genetic parameter estimates for tree height and stem form from ABLUP models with standard errors provided in parentheses

Parameter	Height	Stem form
	Estimate (SE)	Estimate (SE)
Type B genetic correlation (${\hat{r}}_{B}$)	0.785 (0.021)	0.873 (0.021)
Additive genetic variance ($\sigma_{u}^{2}$)	0.203 (0.036)	0.257 (0.015)
Specific combining ability variance ($\sigma_{\mathrm{SCA}}^{2}$)	0.013 (0.003)	0.007 (0.002)
Mean residual variance for fourth-Cycle tests (${\bar{\sigma }}_{e(p)}^{2}$)	0.389 (0.012)	0.934 (0.019)
Mean residual variance for clonal tests (${\bar{\sigma }}_{e(c)}^{2}$)	0.145 (0.002)	0.180 (0.003)
Family-mean heritability ($h_{f}^{2}$)	0.96 (0.003)	0.92 (0.005)
Clone-mean heritability ($h_{c}^{2}$)	0.49 (0.012)	0.51 (0.014)
Narrow-sense heritability ($h^{2}$)	0.31 (0.014)	0.19 (0.010)

### Cross-validation within ACE population

**Figure 3:**
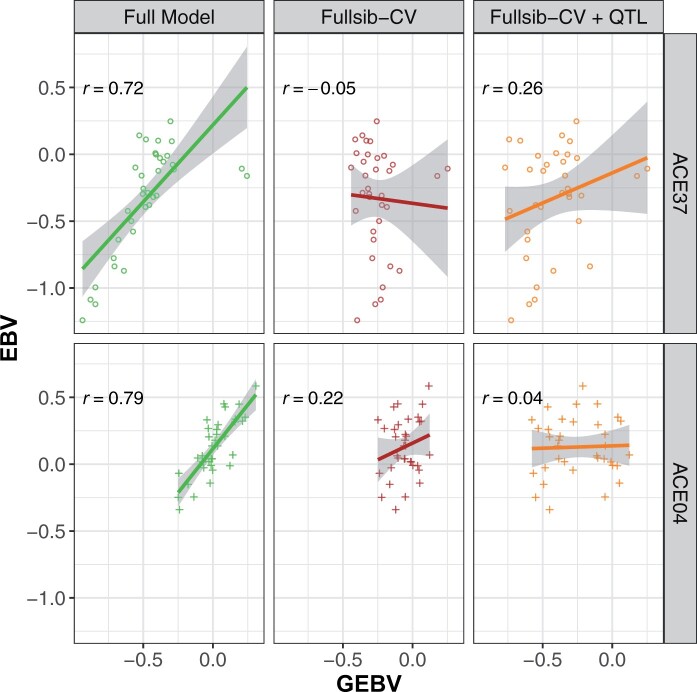
Prediction abilities for tree height (m) of two families segregating for the significant SNP PitaSNP287174 marker on linkage Group 5 for three scenarios. *Left (full model)*: all ACE genotypes are included in the training model; *Center (Fullsib-CV)*: full-sib families ACE37 or ACE04 are removed from the training model used to predict the specific family; *Right (Fullsib-CV+QTL)*: full-sib families ACE37 or ACE04 are removed from the training model, and GEBV are adjusted with the QTL effect on linkage Group 5.

The Random-CV scenario showed contrasting levels of prediction ability for height and stem form, with average prediction abilities of 0.58 and 0.78, respectively (Table 2). Adjustment of height GEBV with the QTL covariate increased prediction ability by only one percentage point in the Random-CV scenarios (not shown). The modest increase in prediction ability using the covariate was likely due to its low minor allele frequency and the variable marker-QTL linkage phase observed in the families segregating for the marker. In the families segregating at PitaSNP287174, adjustment of GEBV with the QTL covariate resulted in large changes to prediction ability. In the case of family ACE37, adjustment with the covariate increased the prediction ability from –0.05 to 0.26 in the Fullsib-CV scenario (Figure 3). Similarly, in family ACE76, the QTL covariate increased prediction ability from 0.36 to 0.38 (data not shown). However, prediction ability for family ACE04 was reduced from 0.22 to 0.04 with the QTL effect, again suggesting that linkage phase between the minor allele at PitaSNP287174 and height-reducing QTL allele varies among families.

**Table 2:** Cross-validation model fit statistics for six scenarios

Scenario	Training/prediction	Prediction ability (*SE*)	Slope (*SE*)	Mean top 10%
(a) Height				
Random-CV	1558/413	0.58 (0.03)	0.73 (0.07)	0.38 (0.02)
Fullsib-CV	2024/39	0.22 (0.18)	0.39 (0.35)	0.19 (0.19)
4C	2063/451	0.29	0.39	0.29
4C Full-Sib	2063/57	0.16	0.16	0.33
4C Half-Sib	2063/29	0.23 (0.13)	0.23 (0.18)	0.17 (0.21)
4C Unrelated	2063/186	0.24	0.56	0.25
(b) Stem form				
Random-CV	1558/413	0.78 (0.01)	0.81 (0.04)	–0.37 (0.04)
Fullsib-CV	2024/39	0.36 (0.16)	0.44 (0.22)	–0.09 (0.33)
4C	2063/451	0.57	0.71	–0.19
4C Full-Sib	2063/57	0.37	0.17	–0.02
4C Half-Sib	2063/29	0.29 (0.27)	0.26 (0.23)	0.07 (0.32)
4C Unrelated	2063/186	0.04	0.07	–0.11

For replicated scenarios, standard errors are provided after each estimate. Random-CV, random approximately fivefold cross validation within the ACE training population with 10 reps; Fullsib-CV, each full-sib family is predicted using a training set lacking any members from that full-sib family, with 51 reps; 4C, 451 fourth cycle trees are predicted using the ACE training population; 4C Full-Sib, fourth cycle trees within one family having full-sib relatives in the ACE training population; 4C Half-Sib, fourth cycle trees within seven families having half-sib relatives in the ACE training population; 4C Unrelated, fourth cycle trees not having direct parental relationships with the ACE training population.

Prediction ability for both traits dropped significantly in the Fullsib-CV scenarios (masking single full-sib families) relative to Random-CV (Table 2). The average prediction ability within 51 families was 0.22 for height and it was 0.36 for stem form. These correlations represent decreases of 62% and 54% from the Random-CV scenario, respectively. The standard errors of prediction ability for both traits showed a large increase in Fullsib-CV relative to Random-CV, indicating more variability in the prediction ability. The addition of the QTL covariate had minimal impact on the mean prediction ability, but did have a significant impact on the families segregating for the marker (Figure 3).

### Individual tree GEBV

**Figure 4:**
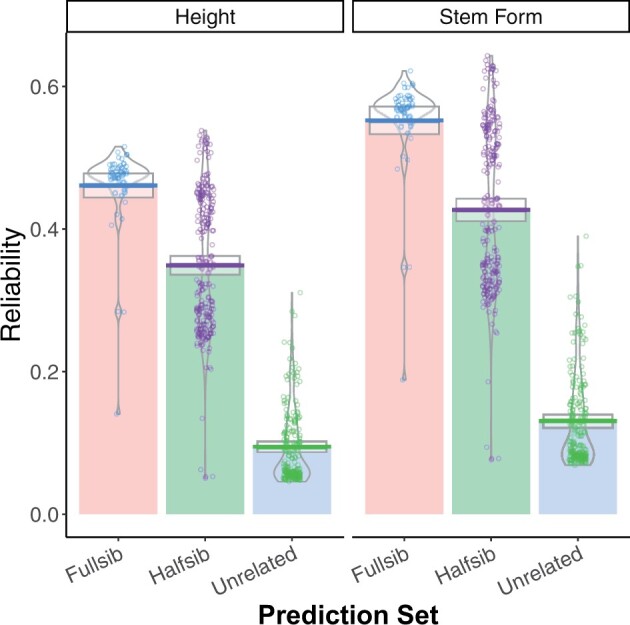
Estimated reliabilities from GBLUP for height (left) and stem form (right) for 451 individual trees. On the *X* axis, three categories of genotypes are presented: fourth-Cycle trees with full-sib relatives in the ACE training population, fourth-Cycle trees with half-sib relatives in the ACE training population, and fourth-Cycle trees without direct relatives in the ACE training population.

**Figure 5:**
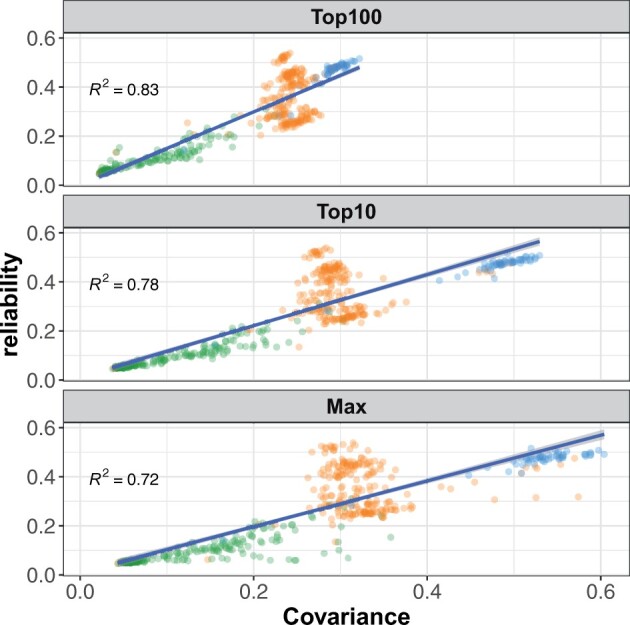
Reliability of individual tree height GEBV is strongly associated with relatedness to the training population. Three types of relationship between training and prediction sets are shown: fullsibs (blue dots), halfsibs (orange dots), and unrelated (green dots). *Top100*: Mean of the top 100 covariances with the training population explains 83% of the variation among reliabilities. *Top10*: Mean of the top 10 covariances with the training population explains 78% of the variation among reliabilities*. Max*: Maximum covariance with the training population explains 72% of the variation among reliabilities.

Reliabilities of individual tree GEBVs were strongly influenced by the degree of relationship with the training population (Figure 4). For tree height, reliabilities were reduced by 18%, 36%, and 82% in the GBLUP model relative to ABLUP for full-sibs, half-sibs, and unrelated trees, respectively (Figure 4). Stem form reliabilities were not significantly reduced for full-sibs, and were reduced by 19% and 75% for half-sibs and unrelated trees, respectively. Reliabilities of GEBV for individual trees were regressed on three different measures of covariance with the training population (Figure 5). The mean of the top 100 covariances between the individual tree and the training population explained 83% of the variation in reliability. The mean of the top 10 covariances and the maximum covariance explained 78% and 72% of the variation in reliabilities, respectively. Variation in the mean of all covariances among genotypes was very low, and explained only a small proportion of the variation in reliability (not shown).

**Figure 6:**
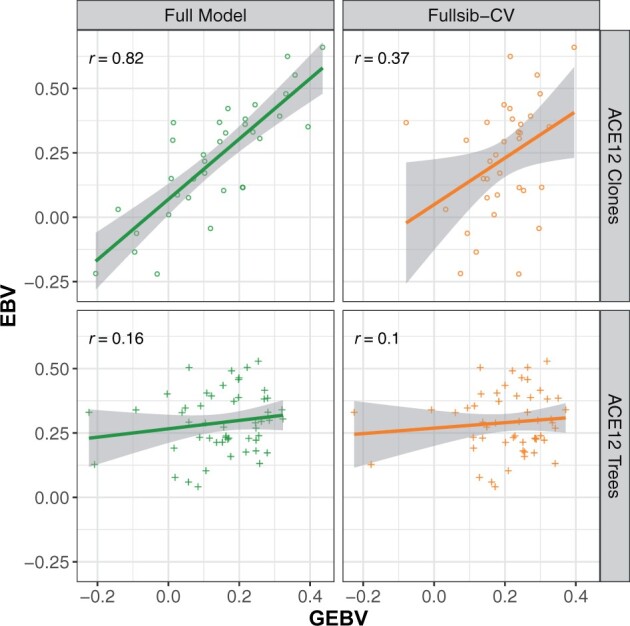
Height GEBV predicted for ACE clones (top row) or fourth-Cycle trees (bottom row) from full-sib family ACE12 using a GBLUP model trained with all ACE genotypes or with a restricted population lacking full-sib relatives (Fullsib-CV). EBV estimated from the animal model are shown on the *Y* axis, while GEBV from GBLUP are on the *X* axis. Prediction ability (r), shown in the upper left of each plot, is the Pearson correlation between GEBV and the corresponding EBV from ABLUP.

Genomic prediction ability for individual trees in fourth-Cycle progeny tests was far lower than for clones within ACE. For tree height, genomic prediction abilities dropped from 0.58 for clones to 0.29 for individual fourth-Cycle trees (Table 2a). A reduction in genomic prediction ability between clones and individual trees was also observed for stem form, although not as large as for tree height (Table 2b). One full-sib family, ACE12, contained both clones and individual fourth-Cycle trees. The Fullsib-CV scenario for this family indicated that genomic prediction ability is reduced significantly for both clones and individual trees when the entire full-sib family was removed from the training set (Figure 6).

The average prediction ability for all 451 individual trees was 0.29 for height and 0.57 for stem form (Table 2). For the set of full-sibs, the prediction ability was 0.16 for height and 0.37 for stem form. For height and stem form, prediction ability within half-sib families averaged 0.23 and 0.29, respectively. The greatest contrast between the two traits was observed for the unrelated prediction set. For height, the prediction ability for individual trees without direct parent relationships to the training population was 0.24 (Table 2a). For stem form, this correlation was 0.04 (Table 2b). The large contrast between the two traits in the unrelated prediction set may have been related to low narrow-sense heritability for stem form in ABLUP (Table 1). For tree height, the narrow-sense heritability was around 32% of the family-mean heritability, but was only ∼19% for stem form. A significant amount of inflation of GEBV relative to EBV was observed, particularly for the fourth-Cycle trees. Slopes of EBV on GEBV were all lower than 1. For clonal genotypes, slopes were significantly reduced in the Fullsib-CV scenario relative to Random-CV. For both tree height and stem form, the mean of the top 10% of EBVs ranked by GEBV suggested that genetic gain could be realized through selection based on GS.

### Linkage disequilibrium

**Figure 7:**
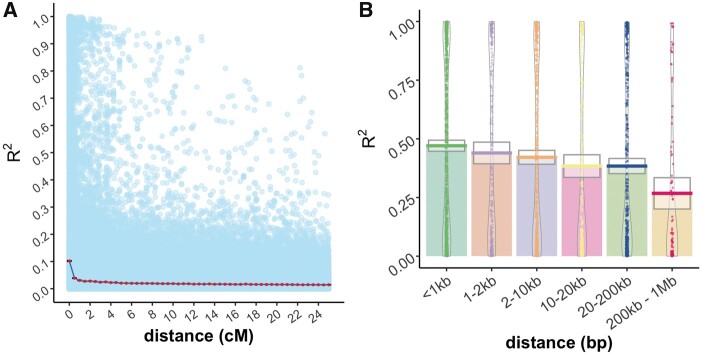
(A) Decay of linkage disequilibrium (R^2^) is shown relative to genetic distance (cM). The decay function is the mean for all pairs of markers with a genetic distance equal to or less than the value on the *X* axis. Pale blue dots in the background are marker pairwise R^2^ values. (B) LD decay is shown relative to physical distance for pairs of markers mapped to the same contigs of the Pita v.2.01 reference genome.

The decay of LD was studied for pairs of markers placed on the Lauer and Isik (2021) consensus genetic map. Pairs of markers on the consensus map located at the same genetic position had a median R^2^ close to 0.1 (Figure 7A). This correlation dropped by more than half with a 1 cM increase in map distance and was close to zero at distances greater than 2 cM. Decay of LD was also studied for pairs of markers located on the same contigs of the Pita v.2.01 reference genome. For pairs of markers < 1 kb distant, the median R^2^ was 0.48; this correlation dropped by 50% within 1 Mb (Figures 6B and 7B).

## Discussion

This study shows the large impact of family structure on empirical prediction ability for tree height and stem form in *P.taeda*. Some recommendations can be made regarding the practical application of GS in tree breeding. First, the inclusion of full-sib relatives of selection candidates in the training population increased the within-family prediction ability for both traits by more than 50% (Table 2). The reduction in prediction ability when full-sib relatives were removed from the training population was severe, even if those relatives constituted < 2% of the total training population size. Second, the impact of large-effect QTL on height varies by family. The assumption of common linkage phase between markers and QTL is not valid, particularly in outbred conifers with large effective population size, large physical genome size, and limited breeding history from their wild source populations (Brown *et al.* 2004; Zimin *et al.* 2014; Isik and McKeand 2019). There may be benefits from models that utilize multi-allelic haplotypes (Hess *et al.* 2017; Sallam *et al.* 2020) or that explicitly model the transmission of QTL alleles from parents to offspring (Sun *et al.* 2016).

Missing heritability for height and stem form was 0.26 and 0.13, respectively. These estimates are informative as to the relative efficiency of GS selection *vs* traditional ABLUP. If the breeder were to select among individual seedling progeny from the same group of families in the training population, the selection accuracy would be around 74% and 87% of what could be obtained if they waited for height and stem form measurements, respectively. Assuming a 50% reduction in cycle time and an equal selection intensity, the annualized genetic gain from GS compares favorably with ABLUP for both traits. However, this advantage depends on close genetic relationships between the training and prediction sets. The selection accuracy decreases as this genetic similarity gets lower.

Prediction abilities in the Random-CV scenario within the ACE clonal population were 0.58 for height and 0.78 stem form, comparing favorably to maritime pine (Isik *et al.* 2016) and white spruce (Beaulieu *et al.* 2014). Since each replicate of Random-CV featured training and prediction sets within the same generation, these prediction abilities are likely higher than would be obtained from cross-generation prediction since marker-QTL linkage phase was consistent between training and prediction sets in Random-CV (Bartholomé *et al.* 2016; Isik *et al.* 2016). Within the ACE clonal population, a ∼60% reduction in prediction ability was observed in the Fullsib-CV scenario relative to the Random-CV scenario for both traits (Table 2). In most GS studies reported in conifers, results from Random-CV are reported as the metric for assessing genomic prediction ability (Resende *et al.* 2012a; Isik *et al.* 2016; Lenz *et al.* 2017; Thistlethwaite *et al.* 2020). Since each prediction set in Random-CV contains individuals from multiple full-sib or half-sib families, Mendelian sampling effects are confounded with family means and the prediction ability appears higher (Werner *et al.* 2020). In this study, by systematically removing each full-sib family from the training population, the prediction ability for Mendelian sampling effects can be partitioned from the prediction ability for family means. In most breeding programs, breeding values for the parents that are intercrossed to produce the GS training and validation populations are known, since they are usually selected using progeny records via ABLUP. The expectation of the family mean is the mid-parent breeding value, which is either already predicted with a high degree of precision or can be estimated from the phenotypic records within the training population. The true utility of genome-wide markers to tree breeding lies in the prediction of Mendelian sampling effects (Werner *et al.* 2020), which would allow large collections of full-sib progenies from a cross to be ranked using genome-wide markers without the need for phenotypic data or progeny testing. The observed variation in marker-QTL linkage phase across families (Figure 2) and rapid decay of LD (Figure 7) suggests that the current marker density (1 SNP/755Kb) may not adequately capture within-family haplotype variation. Likewise, since only 21 parents were intercrossed to produce the ACE training population, the haplotype diversity within the training data may have been inadequate for the prediction of Mendelian sampling effects from one family to another (Werner *et al.* 2020). The rate of LD decay measured for *P. taeda* is similar to other conifers such as *Pseudotsugamenziesii* and *Piceaglauca* (Thistlethwaite *et al.* 2020). The combination of rapid LD decay and exceptionally large genome size (Zimin *et al.* 2014) means that in conifers, higher marker density, and more diverse training populations may be required for within-family selection schemes.

For prediction abilities of the individual fourth-Cycle trees, the impact of relatedness to the training population was more obvious for stem form than height (Table 2). The most significant contrast between the two traits was for the unrelated set, which had an average prediction ability of 0.24 for height and 0.04 for stem form. For tree height, there was little difference between the full-sib, half-sib, and unrelated prediction sets (Table 2a). For stem form, prediction ability was well correlated with the degree of relationship between training and prediction sets, dropping from 0.37 to 0.29 between full-sibs and half-sibs, and from 0.29 to 0.04 between half-sibs and the unrelated set. The sixfold greater prediction ability for height than stem form for the unrelated set is surprising given that stem form had higher reliabilities for all other prediction sets (Figure 4). This result suggests that stem form was more sensitive to the family structure in the training population than tree height, which may point to subtle differences in genetic architecture between the traits. This sensitivity to family structure was also observed in the results from the ABLUP, in which the difference between family-mean and narrow-sense heritability was much greater for stem form than height (Table 1). Since the unrelated set had the lowest average covariance with the training population of all prediction sets (Figure 5), the prediction ability for these trees would have been driven more by trait heritability than covariance with the training population. The narrow-sense heritability of stem form was 50% lower than tree height (Table 1), resulting in lower prediction ability particularly for the unrelated trees. A similar contrast between growth and form was observed in maritime pine; in that species, sampling from the progeny generation improved prediction ability for stem sweep, but not for height (Isik *et al.* 2016). The prediction ability within the half-sib family sets was comparable to the clonal prediction abilities from the Fullsib-CV scenario for height, and around 20% lower for stem form (Table 2). This shows that clonal replication resulted in significant improvement in prediction ability for stem form, but not for height.

For both traits, the reduction in GEBV reliability from trees with full-sib relationships to trees with no parental relationships to the training population was close to 80%. The magnitude of this reduction in reliability between full-sibs and unrelated individuals is similar to that reported in Merino sheep (Clark *et al.* 2012) and slightly lower than white spruce (Beaulieu *et al.* 2014). Variation among GEBV reliabilities were well explained by the elements of **G** (Figure 5). A total of 83% of the variation among reliabilities for fourth-Cycle trees was explained by the mean of the top 100 covariances in the realized genomic relationship matrix. Slightly less variation among reliabilities was explained by the mean of the top 10 covariances and the maximum covariance, respectively. These values are similar to those reported by Clark *et al.* (2012) for Merino sheep, but in the case of *P. taeda*, there was a larger benefit from averaging more covariances, likely due to the nested family structure of the training population.

The large-effect dwarfing allele discovered on linkage Group 5 is the first dominant dwarfing allele reported in a conifer. It warrants further investigation into the mechanisms of height reduction, its impacts on wood quality traits, and the fitness consequences of reduced height in wild populations. A large number of deleterious recessive mutations are thought to exist in wild populations of *P. taeda*, perpetuated by the outbreeding mating habit and high heterozygosity of the species (Franklin 1972). The dominance of the reduced height effect was unexpected given that deleterious alleles are typically recessive (Yang *et al.* 2017), but this presupposes that reduced height carries a fitness cost in wild populations. The finding that a locus bearing a dominant height-reducing allele is still polymorphic after millions of years of natural selection indicates that the selection coefficient for tree height may be small. This exemplifies the tension between natural selection and domestication. For ancient conifers such as *P. taeda*, genomic methods will be invaluable in identifying mutations that have deleterious effects in a breeding population but are neutral in wild populations.

## Conclusions and implications

Based on the findings in this study, some simple guidelines can be developed for the application of GS in conifers. First, the marker panel Pita50K is adequate for among- and within-family selection, but its ability to capture Mendelian sampling variation within families is limited due to rapid LD decay, large genome size, and low diversity in the training population. In order to predict GEBV within full-sib families not represented in the training population, a higher density marker panel and a large and more diverse training population would likely produce significant improvements in prediction ability. Second, the benefit of familial relatedness to the training population was trait-specific. For tree height, prediction ability of unrelated trees was equivalent to the half-sib prediction sets. This situation was reversed for stem form. This shows that some traits are much more sensitive to familial relatedness with the training set than others, which may involve subtle differences in genetic architecture and trait heritability. Finally, the GWAS results reported here suggest that tree height can be influenced by large-effect QTL. Knowledge of trait architecture, represented in this study by the inclusion of a fixed effect covariate for the significant marker, improved prediction ability in some families and reduced it in others (Figure 3). Inference of identity-by-descent status for unobserved QTL alleles using observed marker genotypes is made more difficult by low LD, large wild effective population sizes (Brown *et al.* 2004), and large genome sizes for conifer species (Zimin *et al.* 2014).

## Data availability

File M1.csv (https://doi.org/10.6084/m9.figshare.15023355) contains the genomic marker data for 29135 SNP markers on the Pita50K Affymetrix Array for all 2514 genotyped samples referenced in the manuscript. File M2.csv (https://doi.org/10.6084/m9.figshare.15023316) contains the phased genomic marker data, in “A|G” format, for 8437 SNP markers on the Pita50K Affymetrix Array for 2063 clones within the ACE training population. File phenotypes_ABLUP.csv (https://doi.org/10.6084/m9.figshare.15023343) contains the phenotypic dataset combining the 8 locations from the ACE1 clonal trials with the 18 fourth-Cycle progeny test locations. File pedigree.csv (https://doi.org/10.6084/m9.figshare.15023358) contains the pedigree file used in ABLUP. File phenotypes_GBLUP.csv (https://doi.org/10.6084/m9.figshare.15023508) contains the phenotypic data for genotyped ACE1 clones, as well as an additional 451 rows (lacking phenotypic data) for the individual genotyped fourth-Cycle trees. File Height_BLUE.csv (https://doi.org/10.6084/m9.figshare.15025266) contains the fixed effect estimates for height for all genotyped clones within ACE1. File “SupplementaryTable4.xlsx” (https://doi.org/10.6084/m9.figshare.15023334) contains the genetic map for all markers in M2.csv. File “GBLUP_pedigree.csv” (https://doi.org/10.6084/m9.figshare.15024540) contains the genotype identifiers and row order for the G matrix, to be used by GBLUP.as. File G1.grm.csv (https://doi.org/10.6084/m9.figshare.15024387) is a sparse-formatted realized relationship matrix computed from 29135 markers using Van Raden Method 1, to be used by GBLUP.as. File GBLUP.as (https://doi.org/10.6084/m9.figshare.15024876) is an ASReml job file for running the full GBLUP model as well as Fullsib-CV for height and stem form. File ABLUP.as (https://doi.org/10.6084/m9.figshare.15024705) is an ASReml job file for running pedigree BLUP for the combined ACE + fourth Cycle dataset. All pedigree identifiers have been coded with random alphanumeric strings.

## Supplementary Material

jkab405_Supplementary_Data
