# Supplementary material for: Prediction ability of genome-wide markers in Pinus taeda L. within and between population is affected by relatedness to the training population and trait genetic architecture
Source: G3 (Bethesda). 2021 Nov 25;12(2):jkab405. doi: 10.1093/g3journal/jkab405 (PMC9210318; doi:10.1093/g3journal/jkab405)
Supplement: jkab405_Supplementary_Data [file jkab405_supplementary_data.zip › Suppl/GENETICS-G3-2021-402935-s04.pdf]

Supplementary Table 1. Average effect for significant GWAS marker PitaSNP287174 within four full-sib families and the count of homozygous and heterozygous genotypes within the corresponding family. Average effects in meters are provided in the third column. Statistical significance at the 0.05 level is indicated with an asterisk.

Family	Homozygous / Heterozygous	Average Effect (m)
**ACE 76**	22 / 12	-0.22
**ACE 37**	17 / 18	-1.32*
**ACE 04**	15 / 20	-0.01
**ACE 29**	7 / 6	0.09
