# Supplementary material for: Prediction ability of genome-wide markers in Pinus taeda L. within and between population is affected by relatedness to the training population and trait genetic architecture
Source: G3 (Bethesda). 2021 Nov 25;12(2):jkab405. doi: 10.1093/g3journal/jkab405 (PMC9210318; doi:10.1093/g3journal/jkab405)
Supplement: jkab405_Supplementary_Data [file jkab405_supplementary_data.zip › Suppl/GENETICS-G3-2021-402935-s01.pdf]

		ACE								CYCLE4																	
		T01	T02	T03	T04	T05	T06	T07	T08	2014IFC16	2014IFC17	2014PCL18	2014PCL19	2014RAY20	2014RAY21	2014WES22	2014WES23	2015PCL01	2015PCL02	2015RAY03	2015WEY05	2015WEY06	2016GFC01	2016PCL02	2016RAY03	2016WES04	2016WEY05
ACE	T08	51	51	51	51	51	51	51	51/28	2	2	2	0	0	0	0	0	2	2	2	2	2	2	2	2	2	2
	T07	51	51	51	51	51	51	51/22	22	2	2	2	0	0	0	0	0	2	2	2	2	2	2	2	2	2	2
	T06	51	51	51	51	51	51/28	22	28	2	2	2	0	0	0	0	0	2	2	2	2	2	2	2	2	2	2
	T05	51	51	51	51	51/28	28	22	28	2	2	2	0	0	0	0	0	2	2	2	2	2	2	2	2	2	2
	T04	51	51	51	51/26	26	26	22	26	2	2	2	0	0	0	0	0	2	2	2	2	2	2	2	2	2	2
	T03	51	51	51/28	26	28	28	22	28	2	2	2	0	0	0	0	0	2	2	2	2	2	2	2	2	2	2
	T02	51	51/28	28	26	28	28	22	28	2	2	2	0	0	0	0	0	2	2	2	2	2	2	2	2	2	2
	T01	51/28	28	28	26	28	28	22	28	2	2	2	0	0	0	0	0	2	2	2	2	2	2	2	2	2	2
CYCLE4	2016WEY05	18	18	18	18	18	18	17	18	78	72	73	3	3	4	3	3	85	85	85	85	85	85	79	85	79	85/163
	2016WES04	18	18	18	18	18	18	17	18	64	64	64	64	64	64	61	58	87	87	86	85	86	143	125	162	125/174	163
	2016RAY03	18	18	18	18	18	18	17	18	78	72	73	3	3	4	3	3	85	85	85	85	85	85	79	85/162	79	155
	2016PCL02	18	18	18	18	18	18	17	18	61	61	61	61	61	61	58	55	85	85	85	84	85	143	125/166	162	166	155
	2016GFC01	17	17	17	17	17	17	16	17	78	72	73	3	3	4	3	3	85	85	85	85	85	85/143	79	143	79	136
	2015WEY06	21	21	21	21	21	21	19	21	78	72	73	3	3	4	3	3	85	85	85	85	85/132	78	79	85	79	84
	2015WEY05	21	21	21	21	21	21	19	21	78	72	73	3	3	4	3	3	85	85	85	85/132	131	78	79	84	79	83
	2015RAY03	21	21	21	21	21	21	19	21	78	72	73	3	3	4	3	3	85	85	85/139	132	132	78	79	85	79	84
	2015PCL02	21	21	21	21	21	21	19	21	78	72	73	3	3	4	3	3	85	85/140	139	132	132	78	79	85	79	85
	2015PCL01	21	21	21	21	21	21	19	21	78	72	73	3	3	4	3	3	85/140	140	139	132	132	78	79	85	79	85
	2014WES23	18	18	18	18	18	18	16	18	26	26	26	139	139	139	133	139/120	107	107	106	99	99	51	26	55	26	55
	2014WES22	18	18	18	18	18	18	16	18	32	32	32	159	159	165	165/125	120	107	107	106	99	99	54	32	58	32	58
	2014RAY21	18	18	18	18	18	18	16	18	33	33	33	166	166	172/129	125	120	107	107	106	99	99	57	33	61	33	61
	2014RAY20	18	18	18	18	18	18	16	18	33	33	33	166	166/130	129	125	120	107	107	106	99	99	57	33	61	33	61
	2014PCL19	18	18	18	18	18	18	16	18	33	33	33	166/130	130	129	125	120	107	107	106	99	99	57	33	61	33	61
	2014PCL18	18	18	18	18	18	18	16	18	118	116	118/130	130	130	129	125	120	107	107	106	99	99	57	118	61	118	61
	2014IFC17	18	18	18	18	18	18	16	18	117	117/130	130	130	130	129	125	120	107	107	106	99	99	57	117	61	117	61
	2014IFC16	18	18	18	18	18	18	16	18	124/130	130	130	130	130	129	125	120	107	107	106	99	99	57	124	61	124	61
