# Supplementary figures and images for: Prediction ability of genome-wide markers in Pinus taeda L. within and between population is affected by relatedness to the training population and trait genetic architecture
Source: G3 (Bethesda). 2021 Nov 25;12(2):jkab405. doi: 10.1093/g3journal/jkab405 (PMC9210318; doi:10.1093/g3journal/jkab405)

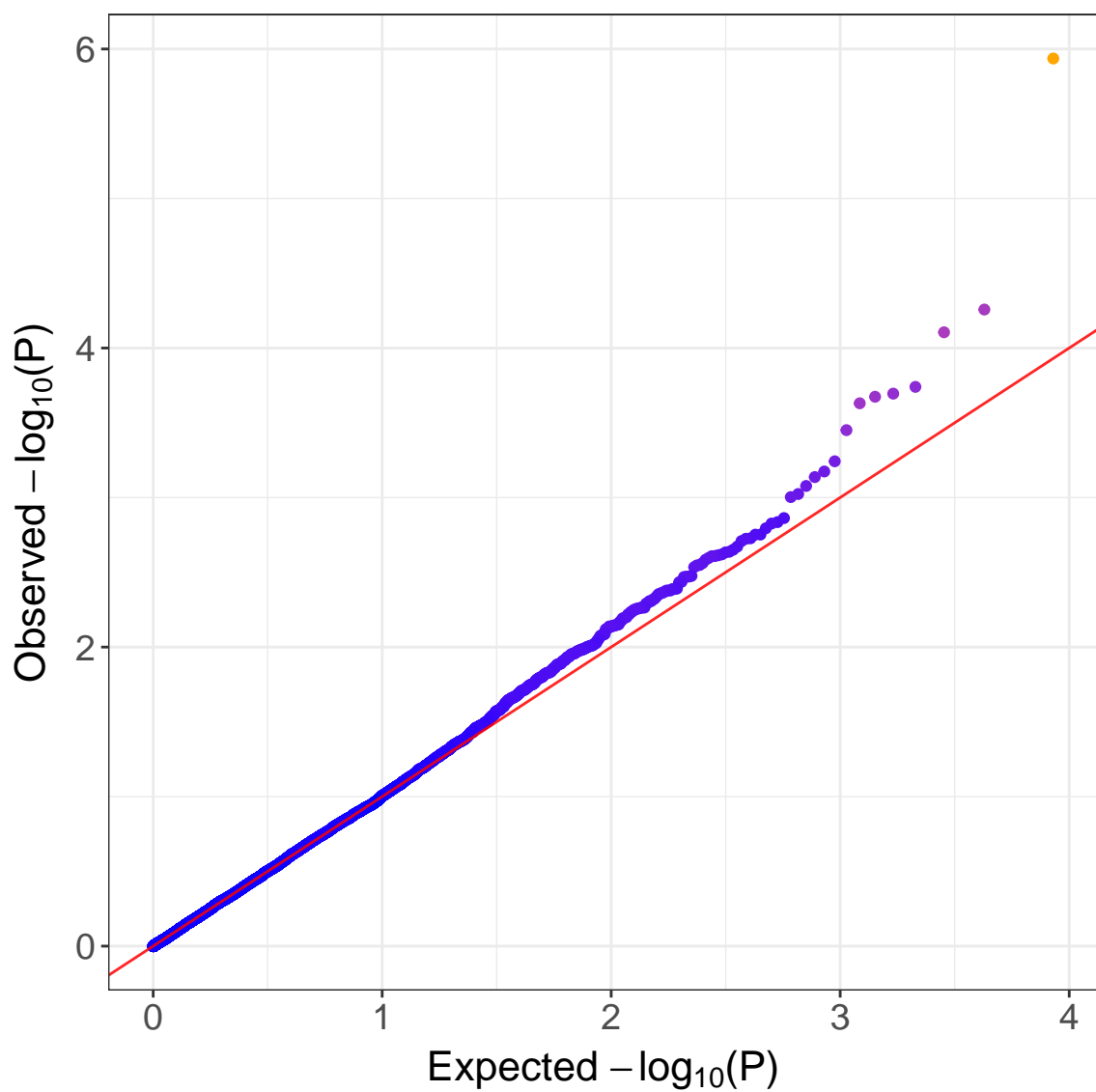

Supplement: jkab405_Supplementary_Data [file jkab405_supplementary_data.zip › Suppl/GENETICS-G3-2021-402935-s02.pdf]

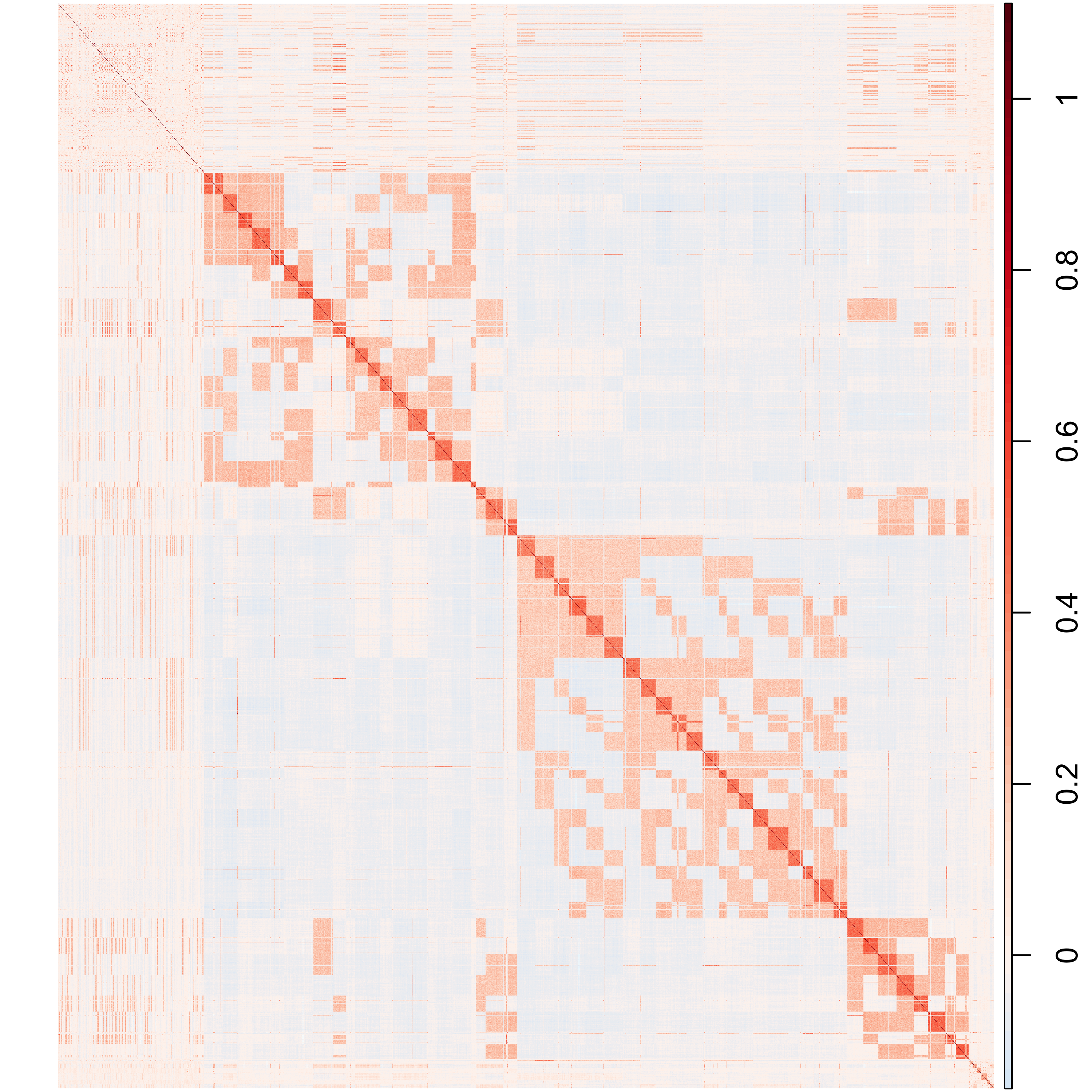

Supplement: jkab405_Supplementary_Data [file jkab405_supplementary_data.zip › Suppl/GENETICS-G3-2021-402935-s03.png]
